# Eighty-Third Annual Meeting of the Medical and Chirurgical Faculty of Maryland

**Published:** 1881-06

**Authors:** 


### EIGHTY-THIRD ANNUAL MEETING OF THE MEDICAL AND CHIRURGICAL FACULTY OF MARYLAND.

Held in Hopkins Hall April, 12th, 13th, 14th, 15th and 16th.

REPORTS OF SECTIONS.

*Section on Surgery.*—After the reports of officers had been read and suitable action taken in reference to them, the report of the Section on Surgery was called for. This report was made through Dr. Oscar J. Coskery, Chairman. He remarked that guided by a rule adopted at a former meeting of this body, he had confined his report to the interesting surgical operations performed, and appliances introduced or extended within the last year by members of the profession in this State and city. Reference was first made to an amputation at the hip joint by Dr. G. G. Rusk. The patient left the hospital well at the end of the third week. Dr. Coskery had again performed a double amputation of both legs, at one sitting, upon a boy, and of right leg and of anterior third of foot, in sister of same boy, for frost bite. Both cases were successful.

Last October Dr. Coskery had removed a large tumor, nearly as large as a man's head, from the neck of a young colored man. This tumor made its appearance twelve years ago. It occupied a position in front of and to the right side of the trachea. It was superficial, hard, knobbed, painless and freely movable from side to side. Under chloroform it was carefully turned out of its capsule. Twenty-two ligatures were put on, and the black heat actual cautery and styptic colloid freely used. Hemorrhage was completely averted by tying two more vessels under the chin, which sprang a leak two hours after the operation. No sutures were used to close the flaps in anticipation of hemorrhage. The sheath of the carotid, with the origin of the facial artery, were bared during the operation. The patient left the hospital on the twenty-first day entirely cured. The tumor was a fibro adeno-enchondroma and weighed three pounds ten ounces. Reference was next made to a case of strangulated hernia in a young married woman, which presented some points of interest Dr. Coskery next exhibited a case of a very peculiar injury to the skull which illustrated the advisability of non-interference unless symptoms demand it. The patient, a sailor, was struck with a large, heavy piece of wood and knocked insensible, and remained so about an hour. When first examined by Assistant Surgeon Goldsborough, at St. Joseph's Hospital, the forehead and right eyelid were so much swollen that no positive diagnosis of the amount of skull implication could be made out, but no positive cerebral symptoms were observed. As the swelling subsided it was found that a portion of the right half of the frontal bone, measuring nearly 1½ to 2 inches, was depressed a quarter of an inch below the general surface of the bone, and that about one inch square of the left half of the frontal was loose and had come forward to nearly as great an extent as the first piece depressed. The patient did perfectly well throughout.

Attention was called to the exceedingly light and easily adjusted apparatus introduced by Prof. Dugas, of Georgia, for fracture of the clavicle. Eight cases treated during the past eight months with this method yielded very good results. The slightness of the apparatus, which consists simply of a triangular piece of cotton and two bandages, is a great advantage.

Dr. Coskery referred to forty cases of stricture of the urethra treated during the past year by Prof C. F. Bevan by the Otis method. In these forty cases ninety-three contractions were divided, seventy-five per cent. of which were located in the anterior four inches of the urethra. The average calibre of the urethra was found to be 30. m. m. F. The relationship between the calibre of the urethra and the circumference of the penis is borne out by the measurements made.

Dr. Coskery again called attention to the modification of the plaster of Paris apparatus decribed at the last meeting of the Faculty. Since then he had used it upon upwards of twenty-five fractures of the leg and nine of the thigh. The result has been good in all cases. This splint possesses the advantage of being lighter than any other apparatus. Dr. Coskery does not claim originality for the idea of "plaster of Paris splints." That honor belongs to Mr. John Croft, of St. Thomas' Hospital, London. Mr. Croft, however, uses thick flannel worked in plaster, while Dr. Croskery uses so much lighter and thinner material.

*Section on Materia Medica.*—The report from this section was read by Dr. A. Atkinson. Attention was directed first to *Crude Petroleum,* the unrefined product from the accumulations on the iron tubes in Pennsylvania and West Virginia. This drug has been found useful in nearly all pulmonary troubles, especially in the cough of phthisis. Its repulsive taste can be improved by making into mass and giving in pill form, as, preferably with Dover's powder or pulv. cubebs, or both combined. In asthma it offers relief. In the oil regions the workers resort to it in all sorts of cough, and without regard to stage of trouble. The crude article is a chocolate colored mass, with odor of coal oil. Crude pertoleum taken in too large doses is apt to cause nausea, with unpleasant eructations. Dr. Atkinson related one case of profound stupor following the swallowing of two ounces of coal oil. The patient was a child 4 years of age. Consciousness returned after two hours, and no bad symptoms followed. Cold douches to the spine and head were resorted to during the stage of stupor, and olive oil freely given. Dr. Atkinson had known intense redness almost erysipelatous to follow the local application of coal oil employed in rheumatism.

*Chian Turpentine* was next considered. This remedy introduced by Dr. Clay, of Manchester, England, as a remedy for cancer of the uterus has not sustained the good repute given to it. A pure article is difficult to obtain. It is more than probable it will fall into disrepute, as condurango and cancer plant did a few years ago. Chian turpentine should come from the island of Chio, in Archipelago. The sample should be of a greenish color, very slightly bitter to the taste, and soon hardened into brittle resin.

*Chaulmoogra Oil* has gained some reputation in treatment of leprosy and other skin affections. It has disappointed many of the English medical officers stationed in Bombay.

*Iodoform* was next considered, and its action on mucous surfaces as a specific in urethritis, chancroids and ulcers generally fully pointed out.

*Cascara Armaga or Honduras Bark* has gained some reputation because of its appetizing and probably alterative powers. In some chronic skin diseases it has been followed by good results, and it is claimed that in constitutional syphilis it exerts a curative effect. Its action is slow, and it is advisable to continue the drug as long as the patient can be induced to take it.

Dr. Atkison called attention to the *Iodide of Starch* as a general antidote for many poisons.

*Dried Blood of the Bullock* is prepared so as to retain the albumen and other nutritious products of the fluid. It perhaps answers a better purpose than the warm blood so much sought after at the Abattoirs of Paris, by consumptives. With glycerine and cognac brandy it forms a pure agent for the speedy supply of blood after hemorrhages, and answers well in marasmus of little children or in chlorotic females without appetite.

Attention was called to the use of *Salicylate of Sodium* in severe cases of purulent cystitis after the benzoic acid had failed to afford relief. No good was experienced until the head became affected by the salt. Its noted power of preserving the purity of the water and preventing decomposition were well attested.

After calling attention at some length to *diuretics, to the acid phosphates, nitro glycerine, mono-bromide of camphor and sclerotic* acid, Dr. Atkinson referred to the value of *boracic acid* as an agent in the treatment of inflammations of mucous membranes and to its specific action in urethritis, to which attention was first directed by Dr. J. Shelton Hill, of this city.

This report on materia medica was quite extended and we have been able only to give a mere outline as indicating rather the subjects presented and not a statement of facts or conclusions.

SECOND DAY'S SESSION.

The Faculty was called to order by the President at 12 M. and the minutes of yesterday's session read and approved. The President announced that Prof. H. Newell Martin, of the biological department of the Johns Hopkins University, would, at the invitation of the Executive Committee, deliver a lecture "*On a Method of Isolating a Mammalian Heart.*" We have copied from "*Notes From the Biological Laboratory,*" of the Johns Hopkins University, the following summary of Prof. Martin's lecture:

"To obtain a mammalian heart isolated from the rest of the body and keep it alive for a time sufficient to allow the examination of the effect of various conditions upon its activity has long been a physiological desideratum. The frog's heart has for years been the subject of minute study, but hitherto the mammalian heart has been a baffling object. It seems to have been forgotten that while the frog's heart is a spongy structure having no arteries of its own, the mammalian heart is a dense organ dependent for its life on a continuous blood flow in its capillaries; and all attempts hitherto made, so far as I know, have been efforts to apply to the mammal the efforts found successful with the frog, with merely the addition of arrangements adapted to keep up the comparatively high temperature at which the mammalian heart normally beats. By working in another way I have recently succeeded in keeping the mammalian heart alive for more than an hour, and beating with perfect rythm and normal force ; the organ is thus made almost as available for study as the heart of a frog. The method adopted is as follows: The animal having been narcotised and the chest opened, the aorta is tied just beyond its arch ; then the trunk which, in the cat, gives origin to the right subclavian and the two common carotids, is ligatured close to its origin, and a canula put in the left subclavian : finally, the inferior and superior venæ cavæ and the azygos vein, and the root of one lung are tied.

Artificial respiration is of course started so soon as the thorax is opened, and kept up henceforth. The course of the circulation is thus:—left auricle, left ventricle, commencement of the aorta, (and along the left subclavian to the canula which is connected with a manometer), coronary system, right auricle, right ventricle, pulmonary vessels of one lung, and then back to the left auricle; in other words, the only section of the systemic circulation left is that through the vessels of the heart itself. Since the physiological actions taking place in the lung are among the best known of all occuring in the body they may be eliminated, and we have practically an isolated and well-working living mammalian heart for study. The nerves going to the heart may be divided if desired, but that is hardly necessary, as the want of blood flow in the nerve centres of the body incapacitates them after a very short time, and they no longer are capable of exerting any influence on the heart. It is possible, however, that changes in the lung vessels may affect the results of experiments made on the heart's work under different conditions, (*e. g.* when defibrinated blood is sent into it from a vein under pressure, or when drugs are administered to it), and an investigation of the nerves, if any, governing the lung vessels must be undertaken as a preliminary to a further study of the direct action of various conditions on the heart's work."

After the conclusion of Prof. Martin's lecture the President introduced Dr. W. T. Sedgwick, of the University, who gave an instructive lecture on "The Study of Blood Pressure in the Coronary Arteries of the Heart." He began by stating that the great anatomist Thebesius, (*Diss. med. inaug. de circ. sang. in corde*, 1708) propounded the theory that the flaps of the semilunar valve of the aorta are pressed against the wall of that vessel during the systole of the heart, and occlude the mouths of the coronary arteries which lie behind them. This view fell into oblivion until it was revived and powerfully supported in recent times by Brucke. The reasoning of the latter is largely theoretical; since the heart is a hollow structure which diminishes its bulk, and, so far as the ventricles are concerned, obliterates its cavity in contraction, he points out that a forcible injection of the heart arteries with blood during the systole of the organ, would tend to make its walls tense and oppose the contraction; while if the coronary arteries received no blood during the ventricular systole but were filled with it during the disatole, the contraction of the heart would not be impeded and its subsequent dilatation would be promoted. Arguing from the general mechanical perfection found in the mammalian body, he concluded that it was probable that the view of Thebesius was correct, and that the semilunar valve flaps were really so placed during the ventricular systole as to prevent blood from entering the proper cardiac arteries; while in disatole the organ had its walls tensely filled with blood and its cavities consequently expanded. The experimental evidence for and against this view cannot be discussed in this brief article; it will suffice to state that prominent physiologists have been hitherto divided on the question, and experiments and anatomical observations have been published on each side; the result being a general belief that the question could only be definitely settled by an observation of arterial pressure in the coronary vessels; if the coronary pulse coincided with that in other arteries Brucke would be wrong; if it alternated with it, he would be right. The difficulty of introducing a canula into a living, beating heart, seems hitherto to have foiled physiological experimentators, and we undertook the task without any very great hopes of success, but we were induced to the attempt by the many important points in the physiology of the mammalian heart and the mechanism of the circulation, which would be rendered available for study should we succeed.

Our experiments were made on dogs, placed very completely under the influence of morphia; after a considerable number of failures we have succeeded in getting on seven or eight animals simultaneous graphic records of mean arterial pressure and pulse beats in the carotid and coronary arteries. The results of a careful examination of these are:

1. The blood pressure in the coronary arteries is comparatively very great, being very little less than in the carotid trunk.

2. The coronary and carotid pulses are practically synchronous in time; there is no trace whatever of an alternation in them. This holds true whether the arterial pressure be high or low, or the heart's rhythm slow or quick; and the minutest feature of the graphic record in the tracing of blood pressure in the carotid is simultaneously and perfectly repeated in that obtained from the coronary artery. Whether the heart's beat be slowed by stimulation of the cardio-inhibitory nerves, or arterial pressure be greatly raised by inducing dyspnœa, the general and sphygmic variations of pressure in the two arteries are perfectly synchronous and similar in form; the record traced from each artery is in its variations an exact duplicate of that obtained from the other.

The results of these experiments prove that for the dog the Thebesius-Brucke view (with a predilection in whose favor we started) is untenable; although the ventricular systole might be conceived to raise pressure in the coronary artery, it is inconceivable that every minute character of the carotid tracing should be synchronously and perfectly reproduced in that from the coronary artery, unless both were due to the same immediate cause, viz: the elevation of arterial pressure in the aorta by the systole of the left ventricle; the mouths of the coronary arteries are therefore not closed by the flaps of the semilunar valve during ventricular systole.

We are now engaged in an examination of the results of stimulation of the accelerator nerves upon the mean pressure in the coronary artery, with the hope of discovering whether these puzzling nerves are not the vasomotor branches controlling the cardiac arteries, but our results on this point are not ready for publication.

A full account of the experiments on which the above statements are based, with reproductions of the tracings obtained, will shortly be published in the *Journal of Physiology.—Maryland Medical Journal.*

